# An Enhancer's Length and Composition Are Shaped by Its Regulatory Task

**DOI:** 10.3389/fgene.2017.00063

**Published:** 2017-05-23

**Authors:** Lily Li, Zeba Wunderlich

**Affiliations:** Department of Developmental and Cell Biology, University of California, IrvineIrvine, CA, United States

**Keywords:** enhancer, transcription factor, embryogenesis, *Drosophila melanogaster*, gene regulation

## Abstract

Enhancers drive the gene expression patterns required for virtually every process in metazoans. We propose that enhancer length and transcription factor (TF) binding site composition—the number and identity of TF binding sites—reflect the complexity of the enhancer's regulatory task. In development, we define regulatory task complexity as the number of fates specified in a set of cells at once. We hypothesize that enhancers with more complex regulatory tasks will be longer, with more, but less specific, TF binding sites. Larger numbers of binding sites can be arranged in more ways, allowing enhancers to drive many distinct expression patterns, and therefore cell fates, using a finite number of TF inputs. We compare ~100 enhancers patterning the more complex anterior-posterior (AP) axis and the simpler dorsal-ventral (DV) axis in *Drosophila* and find that the AP enhancers are longer with more, but less specific binding sites than the (DV) enhancers. Using a set of ~3,500 enhancers, we find enhancer length and TF binding site number again increase with increasing regulatory task complexity. Therefore, to be broadly applicable, computational tools to study enhancers must account for differences in regulatory task.

## Introduction

Nearly every aspect of an organism, from its development to its immune response, is dependent on precise spatiotemporal control of gene expression. This control is mediated by the binding of transcription factor (TF) activators and repressors to stretches of regulatory DNA called enhancers.

Given their role in diverse biological processes, it is not surprising that enhancers vary widely in architecture—length, number of TF binding sites, and the average binding specificity of the TFs that bind them. Enhancers can be ~10–1,000 bps long, with a couple to tens of TF binding sites (Blackwood and Kadonaga, 1998; Yáñez-Cuna et al., 2013). Several theories have been put forth to explain why enhancers are built so differently. For example, differences in evolutionary pressures and TF cooperativity are invoked to explain why many developmental enhancers are robust to rearrangements of TF binding sites within them while some immune-responsive enhancers are intolerant to even point mutations (Thanos and Maniatis, 1995; Kim and Maniatis, 1997; Munshi et al., 2001; Arnosti and Kulkarni, 2005).

Although enhancers vary in architecture, some constraints apply to all enhancers, e.g., an enhancer's need to be distinguishable from the rest of the genome. Because eukaryotic TFs are highly degenerate, TF binding sites litter the genome, and an enhancer can only achieve distinguishability if it consists of a cluster of TF binding sites within a short distance (Wasserman and Fickett, 1998; Berman et al., 2002; Frith et al., 2002; Halfon et al., 2002; Markstein et al., 2002; Rebeiz et al., 2002; Li et al., 2007; Wunderlich and Mirny, 2009; Hardison and Taylor, 2012). Enhancer length, number of TF binding sites, and average specificity of TFs binding an enhancer can be combined in different ways to achieve distinguishability. For example, an enhancer with higher average TF specificity requires fewer TF binding sites than one with lower average TF specificity to be distinguishable from the genomic background.

**Figure 1:**
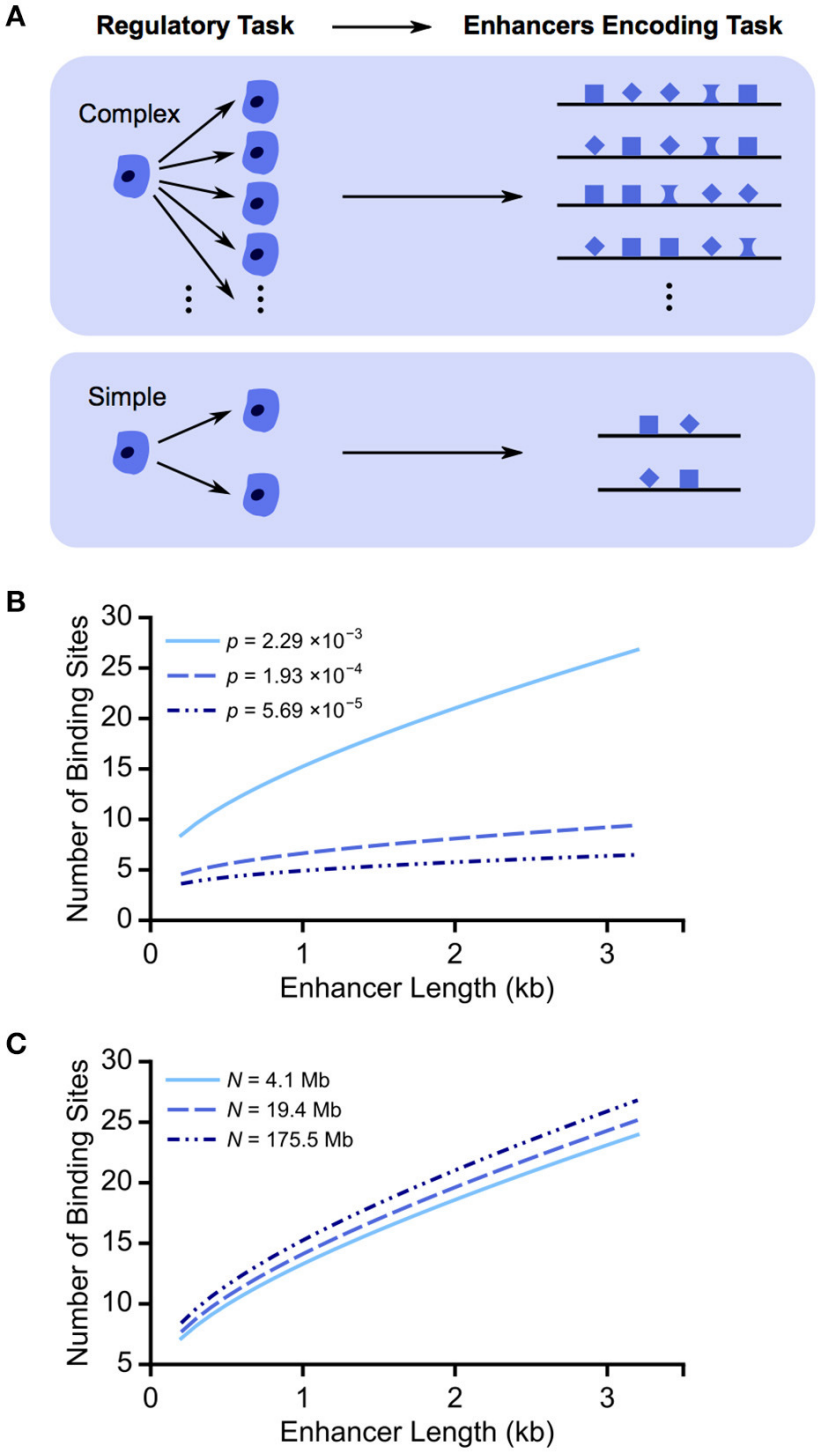
**Regulatory task complexity can shape enhancer length and binding site composition. (A)** We propose that more complex regulatory tasks, e.g., cell patterning decisions, are associated with longer enhancers with more binding sites. More binding sites can be arranged within an enhancer in more ways, allowing for the specification of a wider variety of expression patterns and, therefore, more complex tasks. **(B)** We plot the minimum number of TF binding sites required for enhancers of varying lengths to achieve distinguishability from the genomic background. We show the results for three motif hit probabilities, corresponding to the median, first and third quartiles of *Drosophila* TF binding specificities. As motif hit probability *p* decreases from ~2 in 1 kb (2 × 10^−3^) to ~6 in 100 kb (6 × 10^−5^), an enhancer of the same length requires fewer binding sites to be distinguishable from the background. **(C)** To test the effect of genome accessibility, we plot the minimum number of TF binding sites required for enhancers of varying length in the context of different accessible genome sizes (*N*). Varying the accessible regions of the genome has a minor impact on the trend of numbers of TF binding sites increasing with enhancer length.

We propose that the complexity of an enhancer's “regulatory task”—the process that it controls—is one force that shapes enhancer architecture. In development, task complexity can be defined as the number of cell fates being specified in a set of roughly homogeneous cells at one time. When a cell can be driven to one of many cell fates, the task complexity is high; when a cell is making binary decisions between cell fates, the task complexity is low (Figure 1A). Since cell fate is largely specified by gene expression patterns, the more cell fates being specified, the more distinct expression patterns are needed. To accommodate this need using a limited set of TFs, these enhancers need to contain a larger number of TF binding sites, which allow for more rearrangements and, presumably, more expression patterns. Thus, we propose that enhancers with more binding sites can accommodate higher task complexity. Though intuitive, this proposal has never been verified systematically.

To evaluate this hypothesis, we characterize two sets of enhancers in *Drosophila melanogaster* and analyze the correlation between regulatory task complexity and enhancer architecture. In a set of ~100 early embryonic enhancers, those that pattern the more complex anterior-posterior (AP) axis are longer, have more binding sites, and have lower average TF specificity compared to those patterning the simpler dorsal-ventral (DV) axis. In a set of ~3,500 enhancers active throughout embryogenesis, we find enhancers active early are longer and have more binding sites than those active late, reflecting the general trend that task complexity decreases with developmental time. We conclude that the complexity of an enhancer's regulatory task is one of many forces shaping its architecture.

## Results

To understand the properties required for an enhancer to be distinguishable from the genomic background, we calculate the probability of finding an enhancer with a particular length, number of TF binding sites, and average TF binding specificity (Wunderlich and Mirny, 2009) (Supplementary Material within). As a proxy for TF binding specificity, we use *p*, the probability of finding a “hit” or match to the TF binding motif in the genomic background (see Materials and Methods). Note that a larger *p* corresponds to a lower binding specificity. The probability of finding an enhancer of length *w*, with *k* TF binding sites is:

$$P\left(k\right)=\left(\begin{array}{c} w\\ k \end{array}\right)p^{k}{\left(1-p\right)}^{w\;-\;k}$$

To achieve distinguishability, *P*(*k*) must be less than *1/N*, where *N* is the genome size accessible for TF binding. Thus, the number of required binding sites increases with enhancer length and motif hit probability (Figure 1B). Considering the median, first and third quartiles of all *Drosophila* TF binding specificities, the corresponding number of TF binding sites required in a 1 kb enhancer decreases from 16 to 7 to 5 as TF binding specificity increases (or motif hit probability decreases).

To take into account the compaction of the genome, we consider different values of *N*. We use DNase I hypersensitivity profiles to estimate the accessible regions (Thomas et al., 2011). Whether we use a conservative estimate of accessible regions during development (4.1 Mb), a more relaxed estimate (19.4 Mb), or the entire genome (175.5 Mb) (Thomas et al., 2011; Ellis et al., 2014), the same trends are seen (Figure 1C). For a 1 kb enhancer with binding sites for a TF with relatively low binding specificity, *p* = 2 × 10^−3^, the number of required binding sites increases from 13 to 15 as *N* increases from 4.1 to 175.5 Mb, and thus the number of required binding sites is only weakly dependent on accessible genome size.

To test whether task complexity shapes the characteristics of enhancer architecture, we need a set of enhancers that drive regulatory tasks of different complexities and knowledge of the transcription factors (TFs) that regulate them. The *Drosophila* embryonic AP and DV patterning systems neatly fit these criteria. The AP axis is more complex than the DV axis, with the AP axis consisting of 14 parasegments (Nasiadka et al., 2002) and the DV axis consisting of six germ layers and sublayers (Levine and Davidson, 2005), and therefore the patterning of the AP axis requires enhancers that drive more unique gene expression patterns. Years of work from many groups have identified ~40 principal TFs (Fowlkes et al., 2008; MacArthur et al., 2009) whose binding to ~100 characterized enhancers (Papatsenko et al., 2009) drives AP and DV patterning.

To identify the TF binding sites within these enhancers, we use a computational approach. Though ChIP can experimentally identify TF-bound regions, existing data sets in the *Drosophila* embryo are low resolution, with ~100 base pair peaks (Li et al., 2008; MacArthur et al., 2009; Roy et al., 2010), which are longer than the ~10 bp TF binding sites (Zhu et al., 2011). Therefore, we predict TF binding sites using experimentally measured binding motifs (Stormo, 2015). To select a threshold above which a sequence is deemed a “true” binding site, we develop a principled approach, scoring the aligned sequences used to create the motifs and setting a threshold such that 75% of these aligned sequences are predicted as “true” (see Materials and Methods).

**Figure 2:**
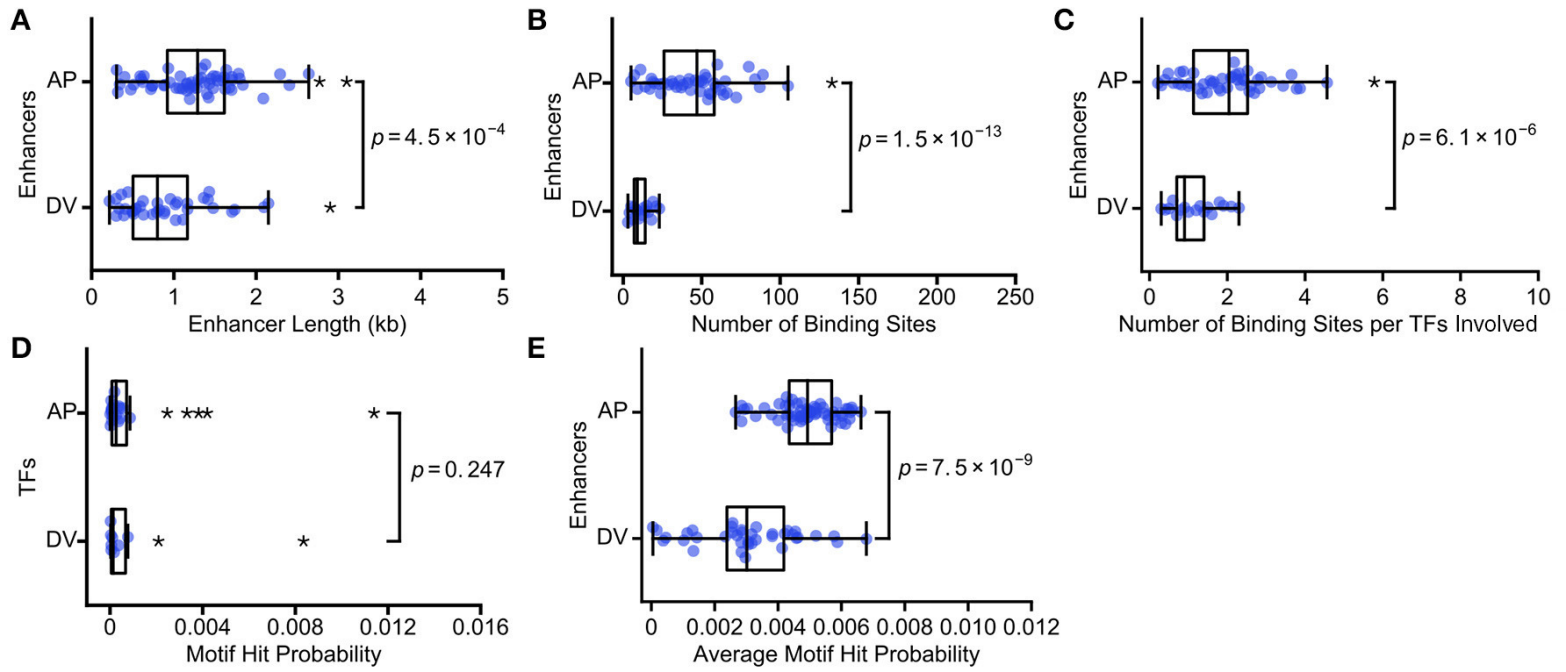
**The more complex AP axis is patterned by enhancers with more TF binding sites**. We show the scatterplots and associated boxplots of **(A)** the length of AP and DV enhancers, **(B)** the number of TF binding sites predicted in AP and DV enhancers, **(C)** the number of TF binding sites normalized by the number of TFs involved, **(D)** the motif hit probability of TFs involved in AP and DV patterning, and **(E)** the average motif hit probability of AP and DV enhancers. These data are consistent with our hypothesis that enhancers carrying out more complex regulatory tasks will have more binding sites, in this case because AP enhancers are both longer and have lower average TF binding specificity. In all box plots, the boxes indicate the lower and upper quartiles, with the line within the box indicating the median. Whiskers extend to 1.5^*^IQR (interquartile range) plus or minus the upper and lower quartile, respectively, and the stars indicate outliers that fall outside the whiskers. *P*-values from Mann-Whitney rank tests are shown.

We analyze 60 AP and 39 DV enhancers, identifying binding sites for 24 AP and 10 DV TFs. Consistent with our predictions, AP enhancers (median length = 1.3 kb) are longer than DV enhancers (median = 0.8 kb; Figure 2A; *p* = 4.5 × 10^−4^; Mann Whitney rank-sum test). AP enhancers also have a larger number of TF binding sites (median = 47) than DV enhancers (median = 9; Figure 2B; *p* = 1.5 × 10^−13^; Mann Whitney rank-sum test). To ensure that the difference is not due to the larger number of AP TFs, we also calculated the number of TF binding sites per enhancer, normalized by the number of TF motifs used to search the enhancer, and find the difference holds (AP median = 2.0, DV median = 0.9; Figure 2C; *p* = 6.1 × 10^−6^; Mann Whitney rank-sum test). AP enhancers also have a higher average motif hit probability (AP median = 4.9 × 10^−3^, DV median = 3.0 × 10^−3^; Figure 2E; *p* = 7.5 × 10^−9^; Mann Whitney rank-sum test), which is a result of differential binding rather than the TFs considered, as the specificity of AP and DV TFs have a similar distribution (Figure 2D, *p* = 0.247; Mann Whitney rank-sum test). This difference is likely driven by the fact that the key TFs that act as morphogens for these axes show markedly different binding specificities, with the key AP axis TFs having low binding specificities and the key DV axis TF having high binding specificity (Figure S1). In summary, we find that the enhancers that encode the lower complexity task of specifying the DV axis are composed of fewer binding sites, as predicted by our hypothesis. The DV enhancers require fewer binding sites because they are both shorter and use more specific TFs than the AP enhancers. Our hypothesis does not require that both enhancer length and motif specificity both differ, though in this case they do.

To determine whether these tradeoffs in enhancer architecture apply to a larger, if less well-characterized dataset, we analyze the Vienna Tile enhancers (Kvon et al., 2014), which drive expression throughout *Drosophila* embryogenesis. To produce this dataset, the Stark lab measured the expression patterns driven by 7,705 enhancer candidates and found 4,480 enhancers that were active during development. Of these enhancers, we consider the 3,580 enhancer candidates that were successfully refined to the putative minimal enhancers using functional genomics (Kvon et al., 2014). To determine the relevant TFs, we match the stages of the active enhancers with the concurrently expressed TFs (Tomancak et al., 2002, 2007; Hammonds et al., 2013).

**Figure 3:**
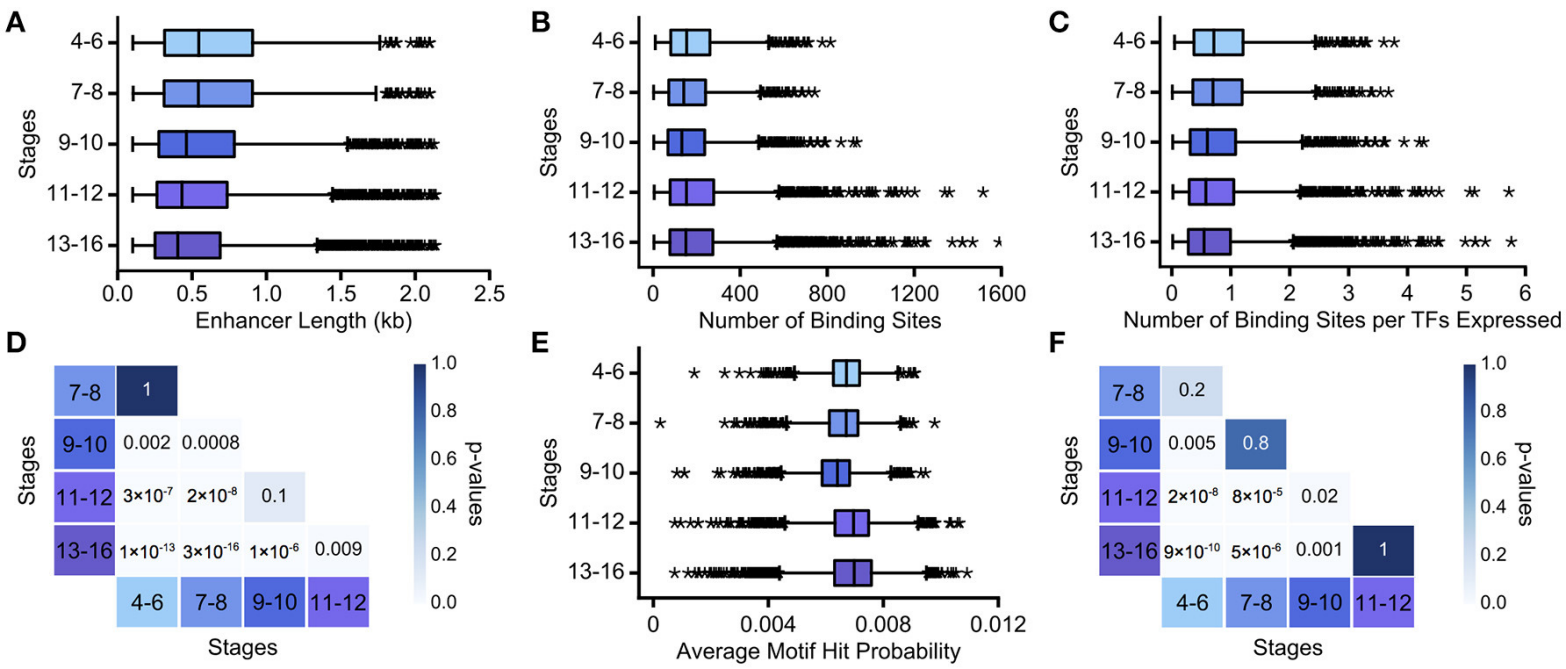
**Decreasing regulatory task complexity over embryogenesis is associated with decreasing enhancer length**. We show boxplots of **(A)** the length of minimal Vienna Tile enhancers, **(B)** the number of TF binding sites predicted in minimal Vienna Tile enhancers, **(C)** the number of TF binding sites predicted in minimal Vienna Tile enhancers normalized by TFs concurrently expressed, and **(E)** the average motif hit probability of minimal Vienna Tile enhancers over developmental stages 4–16. The heatmaps display the Bonferroni-adjusted *p*-values from the Mann-Whitney rank test between **(D)** pairwise distributions of Vienna Tile enhancer length and between **(F)** pairwise distributions of the number of TF binding sites predicted in Vienna Tile enhancers per TFs concurrently expressed. In all box plots, the boxes indicate the lower and upper quartiles, with the line within the box indicating the median. Whiskers extend to 1.5^*^IQR plus or minus the upper and lower quartile, respectively, and the stars indicate outliers that fall outside the whiskers.

We assume that as development progresses, the task complexity decreases, approaching binary decisions between two cell fates. We found that enhancer length monotonically decreases over development (Figure 3A; Table S1). While stages 4–6 and 7–8, and stages 9–10 and 11–12, have very similar length distributions for active enhancers (*p* = 1, *p* = 0.1, respectively; Mann Whitney rank-sum test), all other intervals have significantly different distributions of enhancer length (Figure 3D, *p* < 0.05; Mann-Whitney rank sum test with Bonferroni correction applied). Number of TF binding sites and average motif hit probability, in contrast, do not show a clear trend (Figures 3B,E). However, there is a large increase in the number of TFs expressed in the final two time intervals (see Table S1), and when the number of binding sites is normalized by the number of binding motifs used to search the enhancer, the binding site trend mirrors the enhancer length trend (Figures 3C,F). We also verified that these trends were not unduly influenced by enhancers driving ubiquitous expression patterns (Figure S2, Table S2). Thus, decreasing complexity again is associated with decreasing enhancer length and with decreasing TF binding site number, when normalized appropriately. In this case, there is no clear trend with regards to motif specificity, which, as we note above, is not necessarily at odds with our hypothesis.

## Discussion

We hypothesize that an enhancer's regulatory task complexity shapes its architecture. In the case of *Drosophila* axis patterning, the AP axis has higher task complexity than the DV axis, and accordingly, enhancer length, number of TF binding sites, and average motif hit probability increase with task complexity. In the case of *Drosophila* embryogenesis, where we posit that task complexity decreases over time, enhancer length and binding site number decrease accordingly.

Though the well-characterized *Drosophila* axis patterning systems are ideal for studying how an enhancer's regulatory tasks shape its design (Markstein et al., 2002; Papatsenko et al., 2002; Lifanov et al., 2003; Papatsenko and Levine, 2005; Halfon et al., 2008), the systems still have limitations. For example, autoregulatory enhancers like ftz_up, ftz_zebra, and gt_minus1 (Hiromi et al., 1985; Hiromi and Gehring, 1987; Hoermann et al., 2016) have lower task complexity because they reinforce the expression patterns determined by other enhancers, and therefore, may not be consistent with the observed trends. However, we find these autoregulatory enhancers have parameters that generally fall within the bulk of the distribution. In addition, the enhancer boundaries in this dataset were determined one-at-a-time. However, in the Vienna Tile enhancer set, in which boundaries are determined in a uniform manner, we still find that enhancer length decreases with developmental time and regulatory task complexity.

In contrast to the axis patterning data set, choosing principal TFs for the Vienna Tile enhancers is challenging because there is no consistent annotation of the expression patterns of TFs and enhancers. We match stage-specific expression of enhancers and TFs without considering tissue-specific expression, which impacts both the number of TF binding sites and the average motif hit probability and undoubtedly obscures the clarity of those trends.

We expect that there are many other forces shaping enhancer architecture, like protein-protein interactions between TFs or between TFs and cofactors, and therefore do not expect that regulatory task complexity alone can explain enhancer architecture. Additionally, a particular TF may be employed in an enhancer because it is expressed in the right place at the right time, and not because of its TF binding specificity, though an analysis of the binding specificities of the TFs encoded in the *Drosophila* genome shows that there is a wide distribution of TF specificities that is relatively independent of developmental stage (Figures S3–S6). However, we can make educated guesses about the ways that enhancer architecture may vary depending on regulatory task and use this information to improve our ability to predict and design putative enhancers. As increasingly large sets of enhancers are identified in a variety of biological settings, we will undoubtedly uncover other forces impacting why an enhancer is built in a particular way.

## Materials and methods

### Datasets used in this study

The 60 AP and 39 DV patterning enhancers were collected by Papatsenko et al. (2009) and provided here as Supplementary Data Sheet 1, and in this dataset, we considered the binding of 33 early patterning TFs (Fowlkes et al., 2008; MacArthur et al., 2009). The Vienna Tile enhancer project tested the activity of 7,705 potential enhancers (Kvon et al., 2014). In this analysis, we considered the 3,580 enhancer candidates that were active during embryogenesis and whose boundaries had been refined using DHS regions or CBP/P300-bound and H3K4me1-marked regions. Here, we analyzed the binding of TFs that were concurrently expressed with active enhancers based on the Berkeley *Drosophila* Genome Project *in situ* annotations (Tomancak et al., 2002, 2007; Hammonds et al., 2013). These TF lists are available in Supplementary Data Sheets 2, 3. 51.7% of the AP enhancers and 30.8% of the DV enhancers at least partially overlapped with the Vienna Tile enhancers. The five completely overlapping enhancers showed expression in the same stage (i.e., stages 4–6), except for the DV enhancers pnr and rho (Table S3). Outside of those two, the greatest amount of overlap that did not result in expression at the same stage was 60.5%.

### Transcription factor binding site prediction

Transcription factor (TF) binding sites were computationally predicted using Patser (Hertz and Stormo, 1999) with the position weight matrices (PWMs) from FlyFactor Survey (Zhu et al., 2011). Pseudocounts were added to each element in the PWM in proportion to the intergenic frequency of the corresponding base to a total of 0.01. For those TFs with more than one PWM, the PWM derived from the largest set of aligned sequences was used, except in the case of giant and daughterless.

As no TF binding sites were identified in the set of AP enhancers when using the giant PWM selected using the previous criteria, a switch was made to another available giant PWM in FlyFactor Survey with which binding sites could be predicted. In the case of daughterless, which often binds as a heterodimer and has been identified as one of the key TFs in DV patterning, the PWM that was created using only daughterless and not any heterodimeric partners was used.

For the early patterning dataset, 33 of the 37 TFs (Fowlkes et al., 2008; MacArthur et al., 2009) determined to be principal regulatory factors for AP and DV patterning had available PWMs and were used. PWMs were not available for croc, Stat92E, tsh, or Dad. For the Vienna Tile dataset, a total of 292 TFs that had available PWMs and were expressed during embryogenesis were used.

To determine ln (*p*-value) cutoffs in a systematic manner, the aligned sequences from which the PWMs are derived are scored by Patser, and a 75th percentile ln (*p*-value) was chosen as a cutoff such that 75% of the aligned sequences are considered “true” binding sites. Cutoffs at multiple percentiles were considered, but the overall trends for relative numbers of putative TF binding sites identified remained constant regardless of the chosen cutoff (Figure S7).

Some PWMs were generated from DNase I footprints curated in the FlyReg database (Halfon et al., 2008); aligned versions of these footprints were not directly provided by FlyFactor Survey. The raw sequences were retrieved from FlyReg v2.0 and were aligned when possible. Note that Patser can only score sequences that are the same length or longer than the PWM, so some sequences used to create the PWM have been omitted when determining the percentile cutoffs.

### Evaluating transcription factor specificity

Information content is a measure of TF specificity. To measure information in a motif, we calculated the Kullback-Leibler distance (Schneider et al., 1986; Stormo and Fields, 1998) between the motif and the composition of the intergenic regions of the genome

$$I=\sum_{i\;=\;1}^{L}\sum_{b\in \{A,C,G,T\}}p_{i}\left(b\right)log_{2}\frac{p_{i}\left(b\right)}{q\left(b\right)}$$

where *L* is the length of the motif, *p*_*i*_*(b)* is the frequency of base *b* at position *i* in the motif, and *q(b)* is the frequency of base *b* in the intergenic regions of the genome. Note that *p* = 2^−*I*^ is roughly the probability of a motif hit in the genome for a TF of information content *I* (Berg and von Hippel, 1987). For a set of TF binding sites in an enhancer, an average motif hit probability

$$p_{av}=\frac{\sum_{j}n_{j}2^{-I_{j}}}{n_{\text{total}}}$$

is calculated, where *n*_*j*_ = number of binding sites for TF *j, I*_*j*_ = information content of TF *j*, and *n*_total_ = the total number of TF binding sites in a particular enhancer. If a cluster is composed of sites of *m* different TFs with identical motif hit probability *p*, the probability of finding a cluster of *k* binding sites within *w* bps is

$$P\left(k\right)=\left(\begin{array}{c} w\\ k \end{array}\right){\left(mp\right)}^{k}{\left(1-mp\right)}^{w\;-\;k}$$

Therefore, to characterize the average specificity of TFs employed in a specific enhancer, we choose to compute the average motif hit probability *p*, as opposed to the average information content *I*.

### Quantitation and statistical analysis

Mann-Whitney rank tests were performed to compare all distributions, and the *p*-values were reported. The Mann-Whitney test was chosen because it does not require the assumption that the distributions to be compared are normally distributed. When multiple comparisons were made, the Bonferroni correction was applied.

### Data and software availability

Python code for enhancer architecture analysis is available at GitHub: https://github.com/WunderlichLab/Info_Content.

The axis patterning enhancers originally collected by Papatsenko et al. are available as supplemental data file Supplementary Data 1 (Papatsenko et al., 2009).

## Author contributions

LL and ZW conceived of the study. LL carried out the analysis, and LL and ZW wrote the paper.

## Funding

This work is supported by NIH grants R00HD073191 (to ZW) and T32EB009418 (to LL).

### Conflict of interest statement

The authors declare that the research was conducted in the absence of any commercial or financial relationships that could be construed as a potential conflict of interest.
