# Supplementary material for: An Enhancer's Length and Composition Are Shaped by Its Regulatory Task
Source: Front Genet. 2017 May 23;8:63. doi: 10.3389/fgene.2017.00063 (PMC5440464; doi:10.3389/fgene.2017.00063)
Supplement: Supplementary file 1 [file Table1.PDF]

Table S1. Related to Figure 2; Summary data of minimal Vienna Tile enhancers. The second and third column list the number of active enhancers and TFs expressed during the stages between 4 and 16 listed in column 1. Columns 4-8 list the median values for enhancer length, number of TF binding sites, number of TF binding sites per TF expressed, and average motif hit probability of minimal Vienna Tile enhancers for stages between 4 and 16.

			Medians			
**Stage**	**# Active Enhancers**	**# TFs Expressed**	**Enhancer Length (bp)**	**# TFBSs**	**#TFBSs per TF Expressed**	**Average Motif Hit Probability**
**4-6**	713	216	545	154	0.713	0.00673
**7-8**	878	202	543	141	0.696	0.00671
**9-10**	1530	219	462	132	0.603	0.00641
**11-12**	2307	265	432	153	0.577	0.00697
**13-16**	3334	276	404	151	0.547	0.00699
