# Supplementary material for: An Enhancer's Length and Composition Are Shaped by Its Regulatory Task
Source: Front Genet. 2017 May 23;8:63. doi: 10.3389/fgene.2017.00063 (PMC5440464; doi:10.3389/fgene.2017.00063)
Supplement: Supplementary file 2 [file Table2.PDF]

Table S2. Related to Supplementary Figure 2; Summary data of minimal Vienna Tile enhancers when those driving ubiquitous expression are removed. The second and third column list the number of active enhancers and TFs expressed during the stages between 4 and 16 listed in column 1. Columns 4-8 list the median values for enhancer length, number of TF binding sites, number of TF binding sites per TF expressed, and average motif hit probability of minimal Vienna Tile enhancers for stages between 4 and 16.

			**Medians**			
**Stage**	**# Active Enhancers**	**# TFs Expressed**	**Enhancer Length (bp)**	**# TFBSs**	**#TFBSs per TF Expressed**	**Average Motif Hit Probability**
**4-6**	670	216	545	150	0.694	0.00671
**7-8**	830	202	543	136	0.671	0.00670
**9-10**	1467	219	462	128	0.584	0.00639
**11-12**	2235	265	432	150	0.566	0.00696
**13-16**	3191	276	404	149	0.540	0.00700
