# Supplementary material for: An Enhancer's Length and Composition Are Shaped by Its Regulatory Task
Source: Front Genet. 2017 May 23;8:63. doi: 10.3389/fgene.2017.00063 (PMC5440464; doi:10.3389/fgene.2017.00063)
Supplement: Supplementary file 3 [file Table3.PDF]

Table S3. Related to Figure 2; Overlap between axis patterning and the minimal Vienna Tile enhancers. The first column is a list of the 99 axis patterning enhancers, ordered by the percent overlap (third column) with the minimal Vienna Tile enhancer in the second column. The fourth column indicates whether the minimal Vienna Tile enhancer was expressed concurrently with the axis patterning enhancer during stages 4-6.

AP/DV	Minimal Vienna Tile	Overlap (%)	Minimal Vienna Tile Enhancer Active in Stages 4-6?
eve-37	VT14362.1	100	yes
pnr	VT42370.1	100	no
rho	VT24025.1	100	no
tup	VT9666.2	100	yes
vnd	VT54910.1	100	yes
tld	VT46946.1	97	yes
eve-46	VT14367.1	93.9	yes
h-34	VT27677.1	90.8	yes
slp-ecto	VT1967.1	85.6	yes
kni-cis	VT33934.2	79.7	yes
zen	VT37509.2	79.5	yes
eve-37	VT14361.2	78.7	yes
gt-P	VT55790.2	78.4	yes
gt-1	VT55795.2	75.5	yes
slp-B	VT1971.2	75.2	yes
nub-blst	VT6450.1	75	yes
gt-P	VT55791.1	74.6	yes
eve-46	VT14366.1	74.3	yes
slp1-head	VT1967.2	72.5	yes
pdm2-plus1	VT6483.1	71.4	yes
eve-15	VT14368.1	70.6	yes
prd-1	VT6169.2	64.7	yes
otd-E	VT58874.1	63.1	yes
mes3	VT28266.1	62	yes
pdm2-plus3	VT6484.2	60.5	no
eve-M	VT14368.1	55.6	yes
eve-M	VT14368.1	55.6	yes
h-267	VT27678.1	55.1	yes
prd-plus6	VT6165.1	53.4	yes
otd-E	VT58873.1	52.1	yes
ftz-up	VT37567.1	44.4	yes
sna	VT7920.1	43.9	no
ftz-15	VT37571.1	43.2	yes
vnd-V	VT54905.1	42.2	yes
hnt	VT56875.1	40.6	yes
slp-ecto	VT1966.2	40.5	no

eve-M	VT14367.3	39.2	yes
eve-M	VT14367.3	39.2	yes
vnd-M	VT54907.1	36.3	yes
hnt	VT56876.1	35.5	no
pdm2-plus3	VT6484.1	34.6	no
kni-minus5	VT33936.1	31.9	yes
h-267	VT27679.1	30.4	yes
brk	VT58195.3	30.3	no
ftz-up	VT37567.2	29.3	yes
kni-cis	VT33935.1	29.1	yes
prd-plus6	VT6164.2	28.5	yes
rho	VT24024.2	28.1	no
slp-B	VT1970.2	27.9	yes
h-15	VT27680.2	27.8	yes
gt-minus1	VT55790.2	26.3	yes
gt-23	VT55793.1	25.3	yes
kr-CD1	VT22278.2	24.9	yes
gt-minus1	VT55790.1	24.7	yes
pdm2-plus1	VT6482.1	20.9	no
h-15	VT27680.1	19.9	yes
nub-blst	VT6449.2	18.2	yes
prd-1	VT6170.1	18.1	no
ems-up	VT41284.1	15.8	yes
tll-plus4	VT50157.2	14.4	yes
eve-M	VT14367.2	13.8	yes
eve-M	VT14367.2	13.8	yes
prd-P	VT6169.2	13.6	yes
vnd-M	VT54907.2	12.2	yes
prd-plus6	VT6165.2	11.6	yes
tll-plus4	VT50159.1	11.6	no
slp2-minus3	VT1973.3	11.4	no
slp-B	VT1970.1	11.2	yes
slp-B	VT1971.1	11.2	yes
prd-1	VT6169.3	11	yes
prd-1	VT6170.2	11	no
prd-P	VT6169.1	9.4	yes
nub-plus5	VT6454.2	9.1	no
nub-plus5	VT6455.1	9.1	no
pdm2-plus1	VT6483.2	8.4	yes
pdm2-plus3	VT6483.2	8.3	yes
kni-plus1	VT33934.1	7.6	yes
h-267	VT27677.1	3.9	yes
prd-P2	VT6170.3	0.3	no
