# Supplementary material for: An Enhancer's Length and Composition Are Shaped by Its Regulatory Task
Source: Front Genet. 2017 May 23;8:63. doi: 10.3389/fgene.2017.00063 (PMC5440464; doi:10.3389/fgene.2017.00063)
Supplement: Supplementary file 7 [file Image1.PDF]

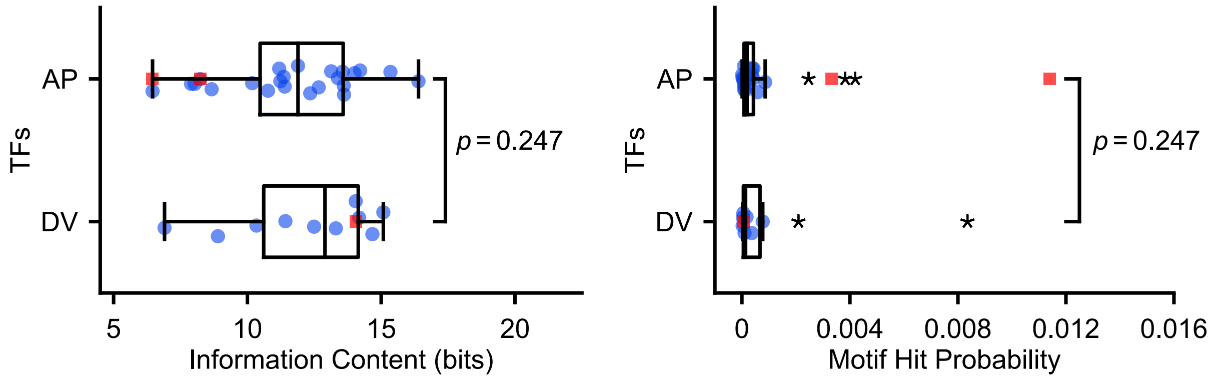

Supplementary Figure 1. The key transcription factors whose gradients set up the anterior-posterior and dorsal-ventral axes fall towards the tails of the information content distribution. We show boxplots of (A) the information content and (B) the motif hit probability of TFs important in axis patterning. The red squares indicate the information content and motif hit probability of the key TFs that act as morphogens to set up the AP and DV axes. These key AP TFs—*caudal* (6.46 bits; 3.32×10^{-3}) and *bicoid* (8.23 bits; 0.0114)—have low information content and high motif hit probability, respectively; whereas, the key DV TF—*dorsal* (14.05 bits; 5.91×10^{-5}) has relatively high information content and low motif hit probability, respectively. In all box plots, the boxes indicate the lower and upper quartiles, with the line within the box indicating the median. Whiskers extend to 1.5*IQR plus or minus the upper and lower quartile, respectively, and the stars indicate outliers that fall outside the whiskers. P-values from Mann-Whitney rank tests are shown.
