# Supplementary material for: An Enhancer's Length and Composition Are Shaped by Its Regulatory Task
Source: Front Genet. 2017 May 23;8:63. doi: 10.3389/fgene.2017.00063 (PMC5440464; doi:10.3389/fgene.2017.00063)
Supplement: Supplementary file 8 [file Image2.PDF]

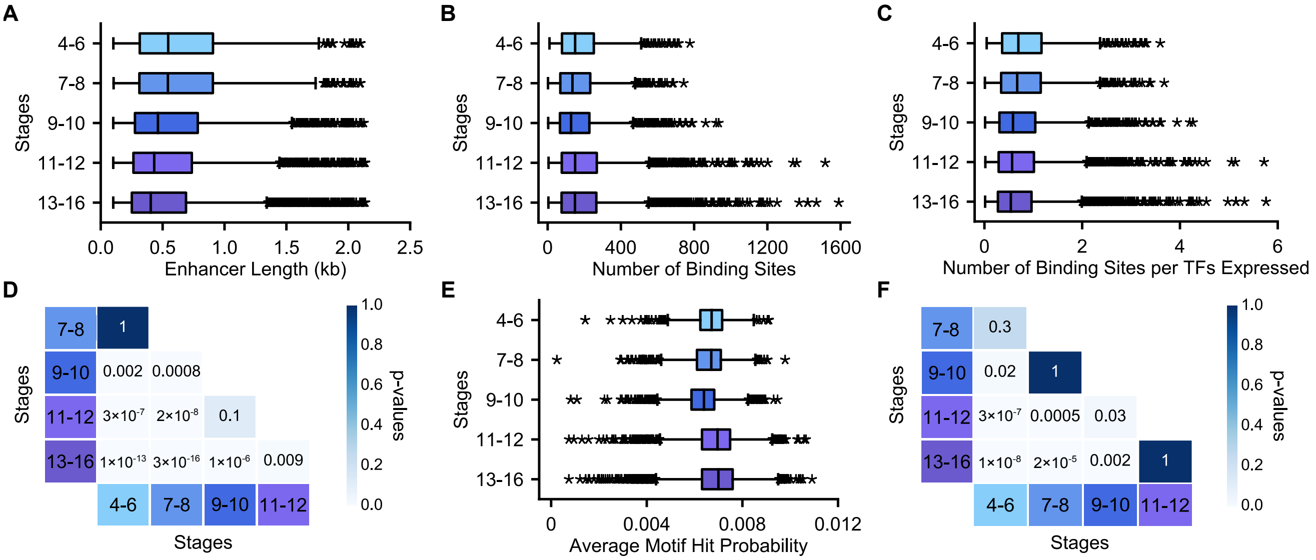

Supplementary Figure 2. When Vienna Tile enhancers that drive ubiquitous expression are removed, the same trends in enhancer architecture are seen. We show boxplots of (A) the length of minimal Vienna Tile enhancers, (B) the number of TF binding sites predicted in minimal Vienna Tile enhancers, (C) the number of TF binding sites predicted in minimal Vienna Tile enhancers per TFs concurrently expressed, and (E) the average motif hit probability of minimal Vienna Tile enhancers over developmental stages 4-13. The heatmaps display the Bonferroni-adjusted *p*-values from the Mann-Whitney rank test between (D) pairwise distributions of Vienna Tile enhancer length and between (F) pairwise distributions of the number of TF binding sites predicted in Vienna Tile enhancers per TFs concurrently expressed. In all box plots, the boxes indicate the lower and upper quartiles, with the line within the box indicating the median. Whiskers extend to 1.5*IQR plus or minus the upper and lower quartile, respectively, and the stars indicate outliers that fall outside the whiskers.
