# Supplementary material for: An Enhancer's Length and Composition Are Shaped by Its Regulatory Task
Source: Front Genet. 2017 May 23;8:63. doi: 10.3389/fgene.2017.00063 (PMC5440464; doi:10.3389/fgene.2017.00063)
Supplement: Supplementary file 9 [file Image3.PDF]

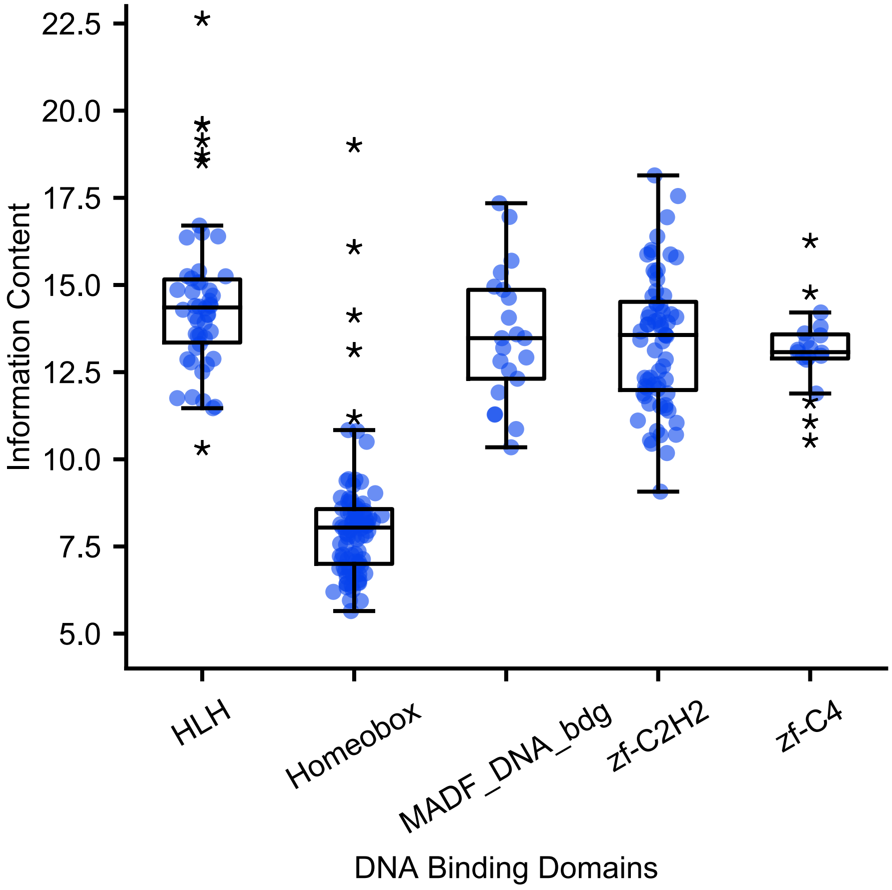

Supplementary Figure 3. The information content of homeobox proteins is generally lower than that of transcription factors with other common DNA-binding domains. We show boxplots of the information content of the TFs by DNA-binding domain, with boxes indicating the lower and upper quartiles and the line within the box indicating the median. Whiskers extend to 1.5*IQR plus or minus the upper and lower quartile, respectively. The stars indicate outliers that fall outside the whiskers. Only those DNA-binding domains that are represented by more than 10 TFs in our sample have been included.
