# Supplementary material for: An Enhancer's Length and Composition Are Shaped by Its Regulatory Task
Source: Front Genet. 2017 May 23;8:63. doi: 10.3389/fgene.2017.00063 (PMC5440464; doi:10.3389/fgene.2017.00063)
Supplement: Supplementary file 10 [file Image4.PDF]

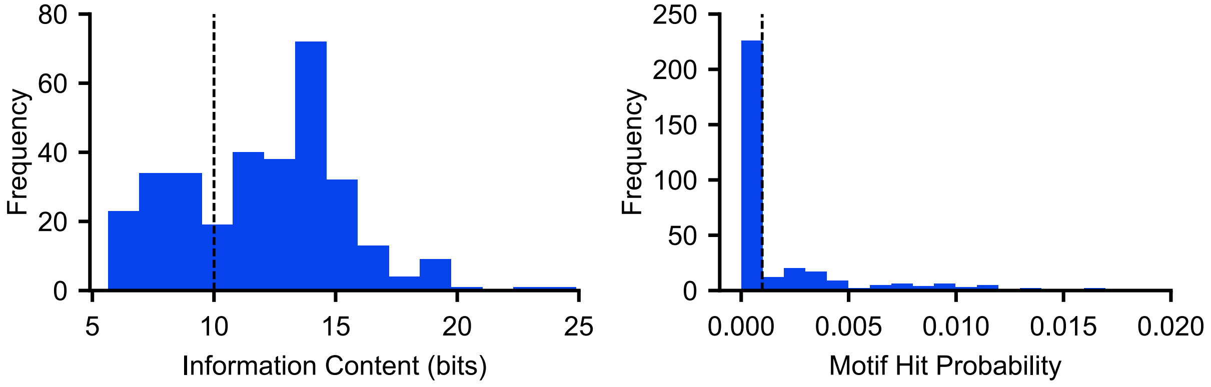

Supplementary Figure 4. The information content of transcription factors fall into a bimodal distribution. (A)We show a histogram of the information content of the *Drosophila* transcription factors with a dashed line indicating a natural separation point between the two peaks. (B) We show a histogram of the motif hit probability of the *Drosophila* TFs with a dashed lined indicating the same point at which the two peaks of information content have been separated. Note that the smaller peak located to the left of the dashed line in (A) corresponds to the smaller peak located to right of the line in this motif hit probability distribution.
