# Supplementary material for: An Enhancer's Length and Composition Are Shaped by Its Regulatory Task
Source: Front Genet. 2017 May 23;8:63. doi: 10.3389/fgene.2017.00063 (PMC5440464; doi:10.3389/fgene.2017.00063)
Supplement: Supplementary file 11 [file Image5.PDF]

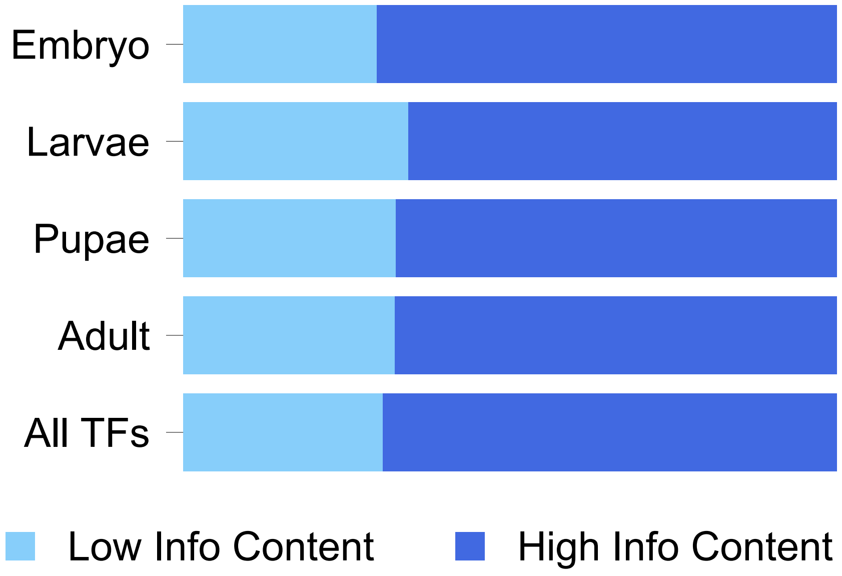

Supplementary Figure 5. The distribution of low and high information content TFs being expressed remains relatively consistent over time. We show stacked bar graphs of the information content of TFs expressed while the flies are embryos, larvae, pupae, and adults, with a bar graph of all TFs for comparison. Each bar has been normalized for the number of TFs expressed at that life stage. Using 10 bits as the natural separation point between the peaks in the bimodal distribution of TF information contents from Supplementary Figure 4, we have divided our TFs into "Low Info Content" and "High Info Content" categories with information contents > 10 bits considered "High." Comparing the fraction of low versus high information content of TFs expressed at any stage compared to that of all TFs shows very little difference, suggesting that any differences we see in the distribution of average information content in enhancers is due to usage of TFs of certain information contents over others.
