# Supplementary material for: An Enhancer's Length and Composition Are Shaped by Its Regulatory Task
Source: Front Genet. 2017 May 23;8:63. doi: 10.3389/fgene.2017.00063 (PMC5440464; doi:10.3389/fgene.2017.00063)
Supplement: Supplementary file 12 [file Image6.PDF]

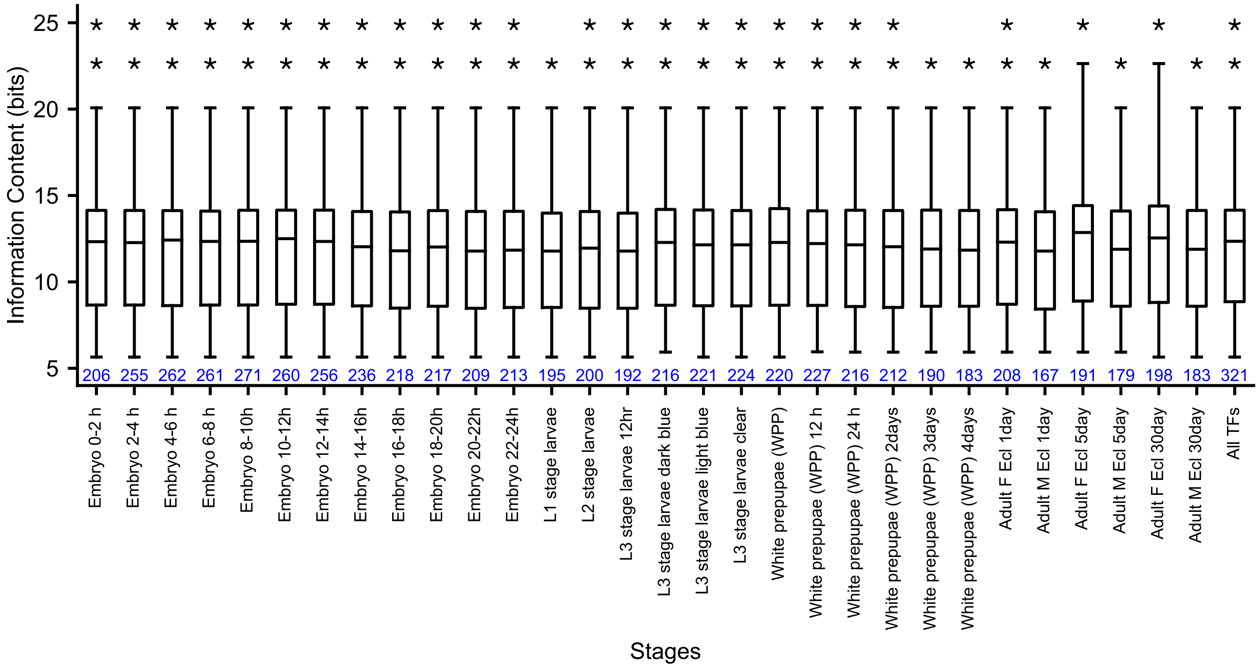

Supplementary Figure 6. The distribution of TF information content remains consistent over time. We show boxplots of the information content of transcription factors being expressed over the lifespan of *Drosophila*, with the last boxplot of the information content of all TFs given for comparison. The boxes indicate the lower and upper quartiles and the line within the box indicating the median. Whiskers extend to 1.5*IQR plus or minus the upper and lower quartile, respectively. The stars indicate outliers that fall outside the whiskers. The numbers in blue above the x-axis indicate the number of TFs expressed at that stage and included in that boxplot.
