# Supplementary material for: An Enhancer's Length and Composition Are Shaped by Its Regulatory Task
Source: Front Genet. 2017 May 23;8:63. doi: 10.3389/fgene.2017.00063 (PMC5440464; doi:10.3389/fgene.2017.00063)
Supplement: Supplementary file 13 [file Image7.PDF]

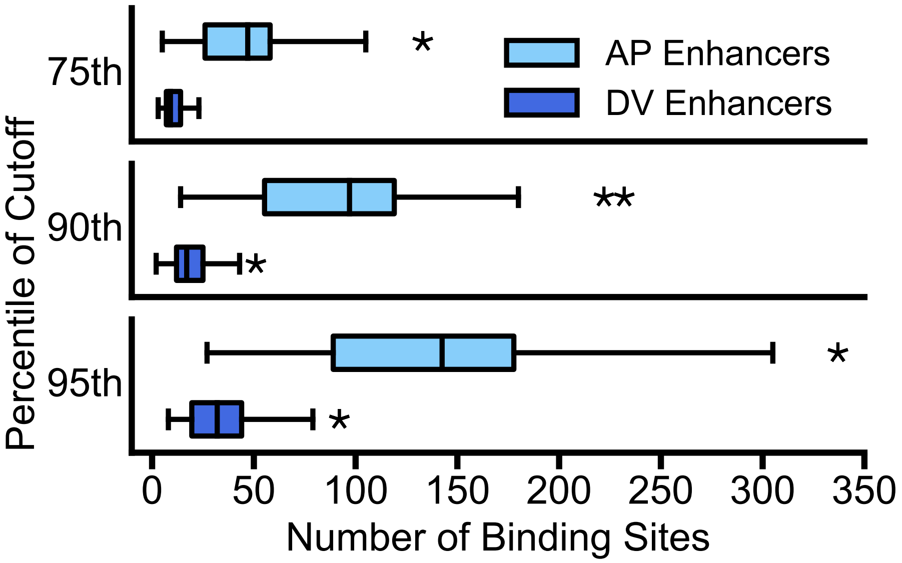

Supplementary Figure 7. Trends in number of predicted binding sites in axis patterning enhancers remain unchanged when cutoffs for "true" binding sites are varied. We show boxplots of the number of TF binding sites predicted in AP and DV enhancers using percentile cutoffs of 75, 90, and 95, with boxes indicating the lower and upper quartiles and the line within the box indicating the median. Whiskers extend to 1.5*IQR plus or minus the upper and lower quartile, respectively. The stars indicate outliers that fall outside the whiskers. P-values calculated by performing Mann-Whitney rank tests on each pair of distributions are less than or equal to 1.5×10^{-13}.
